# Irisin Levels Are Lower in Young Amenorrheic Athletes Compared with Eumenorrheic Athletes and Non-Athletes and Are Associated with Bone Density and Strength Estimates

**DOI:** 10.1371/journal.pone.0100218

**Published:** 2014-06-13

**Authors:** Vibha Singhal, Elizabeth A. Lawson, Kathryn E. Ackerman, Pouneh K. Fazeli, Hannah Clarke, Hang Lee, Kamryn Eddy, Dean A. Marengi, Nicholas P. Derrico, Mary L. Bouxsein, Madhusmita Misra

**Affiliations:** 1 Neuroendocrine Unit, Massachusetts General Hospital and Harvard Medical School, Boston, Massachusetts, United States of America; 2 Pediatric Endocrine Unit, Massachusetts General Hospital for Children and Harvard Medical School, Boston, Massachusetts, United States of America; 3 Division of Sports Medicine, Boston Children’s Hospital, Boston, Massachusetts, United States of America; 4 Department of Biostatistics, Massachusetts General Hospital and Harvard Medical School, Boston, Massachusetts, United States of America; 5 Harris Center, Massachusetts General Hospital, Boston, Massachusetts, United States of America; 6 Endocrine Division, Massachusetts General Hospital and Harvard Medical School, Boston, Massachusetts, United States of America; 7 Department of Orthopedic Surgery, Beth Israel Deaconess Medical Center and Harvard Medical School, Boston, Massachusetts, United States of America; Harvard Medical School, United States of America

## Abstract

Irisin and FGF21 are novel hormones implicated in the “browning” of white fat, thermogenesis, and energy homeostasis. However, there are no data regarding these hormones in amenorrheic athletes (AA) (a chronic energy deficit state) compared with eumenorrheic athletes (EA) and non-athletes. We hypothesized that irisin and FGF21 would be low in AA, an adaptive response to low energy stores. Furthermore, because (i) brown fat has positive effects on bone, and (ii) irisin and FGF21 may directly impact bone, we hypothesized that bone density, structure and strength would be positively associated with these hormones in athletes and non-athletes. To test our hypotheses, we studied 85 females, 14–21 years [38 AA, 24 EA and 23 non-athletes (NA)]. Fasting serum irisin and FGF21 were measured. Body composition and bone density were assessed using dual energy X-ray absorptiometry, bone microarchitecture using high resolution peripheral quantitative CT, strength estimates using finite element analysis, resting energy expenditure (REE) using indirect calorimetry and time spent exercising/week by history. Subjects did not differ for pubertal stage. Fat mass was lowest in AA. AA had lower irisin and FGF21 than EA and NA, even after controlling for fat and lean mass. Across subjects, irisin was positively associated with REE and bone density Z-scores, volumetric bone mineral density (total and trabecular), stiffness and failure load. FGF21 was negatively associated with hours/week of exercise and cortical porosity, and positively with fat mass and cortical volumetric bone density. Associations of irisin (but not FGF21) with bone parameters persisted after controlling for potential confounders. In conclusion, irisin and FGF21 are low in AA, and irisin (but not FGF21) is independently associated with bone density and strength in athletes.

## Introduction

Irisin and FGF21 are novel hormones implicated in the modulation of energy homeostasis [1], and more recently with bone metabolism. A recently discovered myokine and adipokine, irisin has been proposed to be an important mediator of the beneficial metabolic effects of exercise [2]. It is released systemically from skeletal muscle and induces the “browning” of subcutaneous white adipocytes, uncoupling protein 1 (UCP1)-mediated thermogenesis, and increased energy expenditure [2]. Irisin secretion also increases in men who exercise [2], [3]. However, the impact of *chronic* exercise on irisin in over-exercising females has not been examined, and the impact of associated hypothalamic amenorrhea is unknown.

FGF21 is secreted into the circulation from the adipocytes and liver, and is expressed in fat, skeletal muscle and the pancreas. FGF21 regulates carbohydrate and lipid metabolism, resulting in improved glucose homeostasis and lipid parameters, and reduces body weight in animal models. Like irisin, FGF21 promotes conversion of white to beige adipose tissue, activation of UCP1-driven thermogenesis and energy expenditure, although this may represent an autocrine/paracrine rather than endocrine effect [4], [5]. A positive relationship between FGF21 and physical activity has been described [6], and initiation of an exercise regimen in sedentary young women leads to increased FGF21 [7].

In addition, both irisin and FGF21 have been implicated in bone metabolism. Brown adipose tissue is an independent predictor of bone density in women [8], and the volume and activity of brown adipose tissue is positively associated with total and cortical bone cross-sectional area in young children and adolescents [9]. In rodent models, irisin increases trabecular and cortical thickness as well as trabecular density through increased osteoblast activation and inhibition of RANKL mediated osteoclastogensis [10]. Effects of FGF21 on bone are still being defined, with one study demonstrating deleterious effects on bone through inhibition of osteoblastogenesis in favor of adipogenesis [11] whereas a study in adult females reported a positive association between FGF21 and bone density [12] supported by *in vitro* data [13]. Thus irisin and FGF21 may impact bone both through induction of brown adipogenesis, as well as through direct effects. Data are lacking regarding associations of irisin and FGF21 with bone parameters in athletes and non-athletes, and the impact of a hypogonadal state (in athletes with functional hypothalamic amenorrhea) on these associations.

Although exercise may increase irisin and FGF21 levels in healthy individuals, we hypothesized that levels of irisin and FGF21 would be low in amenorrheic athletes, signaling an adaptive response to an overall state of energy deficit. In addition, we hypothesized that irisin and FGF21 levels would be positively associated with measures of bone density, structure and strength in athletes and non-athletes.

## Subjects and Methods

### Subjects

We studied 85 adolescent women [38 amenorrheic athletes (AA), 24 eumenorrheic athletes (EA) and 23 non-athletes] between 14–21 years of age enrolled in an ongoing study, all of whom were >85% ideal body weight based on the 50^th^ percentile for BMI for age. Clinical characteristics of a subgroup of these women have been previously reported [14], [15], [16], [17]. However, levels of irisin and FGF21, and the relationship between irisin and FGF21 levels and measures of bone metabolism have not been previously described. All study participants were recruited from the community through advertisements and referrals from healthcare providers. We defined ‘amenorrhea’ as absence of menses for 3 months in a 6-month period of oligo-amenorrhea (cycle length greater than 6 weeks) or absence of menses at ≥15 years (upper limit of normal for menarche), after other causes of amenorrhea were ruled out. Duration since last menses in AA (median and interquartile range) was 165 (24–360) days. We defined eumenorrheic athletes as those who had had ≥9 menses in the previous year. Three athletes (all less than 17 years old) had not attained menarche, and other causes of menarchal delay (other than excessive exercise) were ruled out in these athletes.

Inclusion criteria for athletes included ≥4 hours of aerobic weight-bearing activity or ≥20 miles of running weekly for the preceding 6 months. Inclusion criteria for non-athletes included <2 hours per week of weight-bearing activities. Per study design, no subject met criteria for anorexia nervosa at study enrollment, although 34% of AA and 4% of EA had a past or current history of some form of disordered eating behavior. The racial distribution included 81.0% White or Asian, 13.1% Mixed and 6.0% Black, while the ethnic distribution included 95.2% non-Hispanic and 4.8% Hispanic. Race and ethnic distribution did not differ significantly across groups.

Subjects were recruited from local area high schools and colleges and referrals from pediatricians, adolescent medicine physicians, endocrinologists, nutritionists and sports medicine physicians. All subjects who met inclusion criteria were included in the study in a consecutive fashion.

### Ethics Statement

The study was approved by the Institutional Review Board (IRB) of the Partners HealthCare system (Protocol 2009-P-000353**)**. Per IRB guidelines and using IRB approved consent and assent forms, (i) informed written consent was obtained from subjects ≥18 years old and parents of subjects <18 years, and (ii) informed written assent was obtained from subjects <18 years.

### Study Design

Subjects were seen in the Clinical Research Center of Massachusetts General Hospital. The screening visit included a history and physical examination, and laboratory evaluations to rule out conditions other than excessive exercise accompanied by inadequate caloric intake that may cause hypothalamic amenorrhea. We thus ruled out hyperprolactinemia, primary ovarian failure, polycystic ovarian syndrome (PCOS) and thyroid dysfunction.

Qualifying subjects underwent a history and physical examination, assessment of hours per week of physical activity (averaged over a year) and measurement of fasting resting energy expenditure using the VMAX Encore 29 metabolic cart (Viasys Healthcare, Carefusion; San Diego, CA) [18], [19]. Subjects did not alter their usual food intake in the days preceding the study visit. Similarly, exercise activity on the days preceding the study visit was not curtailed to allow for usual levels of physical activity. However, fasting morning blood for irisin and FGF-21 levels was drawn before any exercise activity. EA and non-athletes were assessed in the early to mid follicular phase of their cycles (based on menstrual history). All subjects had assessment of (i) body composition and areal bone density (spine, hip and whole body) using dual energy x-ray absorptiometry (DXA), and a subset were assessed for (ii) bone microarchitecture and estimates of strength at the distal radius (site of non-weight bearing bone) using high resolution peripheral quantitative computed tomography (HRpQCT) and finite element analysis (FEA) using published methods [14], [15], [16]. FEA-derived estimates of failure load using these methods are strongly correlated (r^2^ = 0.75) with experimentally measured failure loads that produce Colles’ fractures in human cadaveric radii. HR-pQCT data were acquired on a single instrument by one operator, who performed standard evaluations (periosteal contouring). All finite element analyses (endosteal contouring) were also performed by one blinded investigator [15].

### Biochemical Analysis

We used an ELISA to measure irisin (Adipogen; Liestal, Switzerland; intra-assay coefficient of variation (CV) 6.9%, inter-assay CV 9.07%, detection limit 0.001 mcg/mL) and FGF21 (R&D Systems; Minneapolis, MN; intra-assay CV 3.4%, inter-assay CV 7.5%, detection limit 4.67 pg/mL). For the irisin assay, the laboratory evaluated thee different assay kits on the number of standards (assay range), sample type (serum or plasma), sample volume, sample dilution, assay range, sensitivity, assay time and cost. The Adipogen assay was chosen because it had the greatest detection range (0.001–5 ug/mL), required the least volume of sample, and was the most sensitive (0.001 mcg/mL). This was important given the limited studies of irisin in the pediatric age group, and some uncertainty regarding expected values in a pediatric population. Of note, this assay reports irisin levels that are an order of magnitude higher than the Phoenix Pharmaceuticals assay [20]. All samples were processed immediately and stored at −80 degrees Celsius until they were analyzed to minimize peptide degradation, and contrary to one study that demonstrated inverse associations of irisin levels with duration of sample storage [21], we found no such inverse association of irisin or FGF-21 levels with duration of storage. In fact, another study found no impact of repeated freeze thaw cycles on irisin levels [22], suggesting relative stability of this peptide hormone.

A chemiluminescent immunoassay was used to measure 25-hydroxyvitamin D (DiaSorin, Stillwater, MN; intraassay CV 2.9–5.5%, interassay CV 8%; sensitivity 4 ng/ml). A subset of at least 55 subjects were assessed for P1NP, CTX, testosterone, SHBG and estradiol levels. We used an RIA to measure P1NP (Orion Diagnostics, Espoo, Finland; intra-assay CV 3.5–5.3%, inter-assay CV 3.6–5.4%), lower limit of detection 0.7 ng/ml), and an IRMA to measure CTX (Immunodiagnostics Systems, Fountain Hills, AZ; intra-assay CV 5.2–6.8%, inter-assay CV 5.6–7.4%, lower limit of detection 0.02 ng/ml). Testosterone was measured by RIA (Diagnostic Products Corp, Los Angeles, CA; intra-assay CV 5·1–9·8%, inter-assay CV 8.2%, limit of detection 12 ng/dl), SHBG by IRMA (Diagnostic Products Corp, intra-assay CV 2·8–5·3%, inter-assay CV 8.2%, limit of detection 3 nmol/l), and estradiol by ultrasensitive ELISA (ALPCO Diagnostics, Salem, NH; intra-assay CV of 6.36% and inter-assay CV of 7.6%, minimum level of detection 1.4 pg/mL). Free androgen index (FAI) was calculated using the following formula: [total testosterone (nmol/l)×100]/SHBG (nmol/l) [23]. All blood samples were spun immediately after collection, and the serum separated and stored in −80 degree Celsius freezers until analysis in duplicate.

### Statistical Analysis

JMP Statistical Discoveries (version 10.0; SAS Institute, Inc., Cary, NC) was used for statistical analyses. Our sample size had >80% power for detecting a significant difference between AA and control groups for irisin and FGF21 respectively at an alpha level of 0.05 based on published data [24], [25]. Clinical characteristics, hormone levels and bone parameters were compared across the three groups (AA, EA and non-athletes) using analysis of variance (ANOVA). Irisin and FGF21 levels were logarithmically transformed prior to analyses to approximate a normal distribution. When the overall ANOVA had a significant p-value, the Tukey Kramer test was used to compare differences between groups while controlling for multiple comparisons. For non-parametric comparisons of three groups, we used the Kruskal-Wallis test followed by the Steel-Dwass method for between group comparisons.

We used Pearson or Spearman correlations (depending on data distribution) to determine associations of hormones with bone measures, body composition and REE. Because irisin is a myokine, associations of irisin with these measures may differ in chronically exercising athletes compared with non-athletes. In addition, amenorrhea may modify associations of irisin with various parameters within athletes. For these reasons, we performed exploratory analyses of associations of irisin and FGF21 not only for the group as a whole, but also within AA, EA, all athletes (AA+EA), and non-athletes. Multivariate least-square analyses were constructed to control for confounders and to test interactions between subject group and hormone levels. Significance was defined as a two-tailed p-value <0.05. Data are reported as mean ± SD.

## Results

### Subject Characteristics

Subject characteristics are presented in Table 1. AA were slightly older than the other groups, although bone age and Tanner stage did not differ among groups. Mean duration since the last menstrual cycle in AA was 9.7±2.4 months, and menarchal age was older in AA than in EA and non-athletes. BMI was lower in AA than EA. Total fat mass was lower in AA than EA and non-athletes, and lean mass was higher in EA than in non-athletes. Percent body fat was lower in both groups of athletes versus non-athletes and in AA compared to EA. By design, hours of weekly exercise were higher in AA and EA than non-athletes (with no difference between AA and EA). Resting energy expenditure (REE) was higher in EA than AA and non-athletes. After controlling for lean mass, resting energy expenditure remained higher in EA than AA. REE/lean mass was lowest in AA.

**Table 1:** Clinical characteristics of amenorrheic athletes (AA), eumenorrheic athletes (EA) and non-athletes (NA).

	AA N = 38	EA N = 24	NA N = 23	ANOVA P-value	AA vs. EA P-value	AA vs. NA P-value	EA vs. NA P-value
**Age (years)**	19.3±2.0	18.0±2.0	19.1±1.7	**0.04**	**0.03**	0.86	0.16
**Bone age (years)**	17.4±1.0	17.2±1.2	17.5±1.1	0.49	0.60	0.91	0.50
**Tanner stage distribution (T2, 3, 4, 5)**	2.6%, 10.5%, 15.8%, 71.1%	0%, 4.2%, 20.8%, 75.0%	0%, 8.7%, 0%, 91.3%	0.31	0.66	0.17	0.06
**Age at menarche (y)**	14.0±1.8	12.8±1.4	12.2±1.5	**0.0004**	**0.02**	**0.0004**	0.45
**Days since last menses**	165 (24–360)	-	-	-			
**BMI (kg/m^2^)**	20.3±2.2	22.4±2.3	21.5±2.5	**0.005**	**0.004**	0.15	0.43
**Height (cm)**	165.7±6.7	165.7±8.2	162.6±7.2	0.23	0.98	0.37	0.35
**Fat mass (kg)**	11.7±3.7	15.1±4.4	16.0±4.9	**0.0004**	**0.009**	**0.001**	0.79
**Lean mass (kg)**	43.5±5.7	46.3±7.7	40.5±4.4	**0.006**	0.18	0.15	**0.004**
**Percent body fat**	20.3±5.1	23.5±3.8	27.0±5.2	**<0.0001**	**0.03**	**<0.0001**	**0.04**
**Hours/week of exercise**	10.4±5.6	11.0±4.9	0.8±0.7	**<0.0001**	0.87	**<0.0001**	**<0.0001**
**Resting energy expenditure (calories)**	1247±183	1444±218	1255±193	**0.0005**	**0.0007**	0.99	**0.004**
**REE/lean mass (cal/kg)**	28.9±3.8	31.6±5.3	31.2±4.9	**0.04**	**0.06**	0.13	0.95
**History of fractures**	50%	16.7%	8.7%	**0.0007**	**0.01**	**0.0009**	0.67
**25(OH) vit D (ng/ml)**	38.3±12.5	30.5±13.8	21.1±7.8	**<0.0001**	**0.04**	**<0.0001**	**0.03**
**Irisin (mcg/ml)** §	3.20 (2.58–3.53)	3.85 (3.50–4.80)	3.60 (3.30–4.20)	**0.0004**	**0.001**	**0.009**	0.83
**FGF21 (pg/ml)** §	54.5 (32.3–102.3)	94.5 (60.3–189.8)	132.0 (109.0–230.0)	**0.0001**	**0.02**	**<0.0001**	0.26

Mean ± SD or Median (Interquartile Range); FGF21: fibroblast growth factor 21; AA: amenorrheic athletes; EA: eumenorrheic athletes; NA: non-athletes.

§ P values reported for log converted data.

Bone parameters are shown in Table 2 and have been previously reported for a subset of 50 subjects [14], [15], [16]. Spine BMD Z-scores were lower in AA than EA, whereas hip and whole body BMD Z-scores were highest in EA. Trabecular and cortical volumetric BMD (vBMD), cortical thickness, stiffness and failure load were lower and cortical porosity higher in AA than in non-athletes, and percent cortical area was lower in both groups of athletes compared with non-athletes. Differences among groups remained significant for most parameters after controlling for age, and for age and race (Table 2). Levels of P1NP (a bone formation marker) and CTX (a bone resorption marker) were available for a subset of 55 subjects. P1NP levels did not differ across groups (88.1±37.9, 98.4±55.2 and 83.8±38.3 ng/ml in AA, EA and non-athletes respectively, p = 0.60). CTX levels trended higher in the athletes (1.06±0.33, 1.16±0.39 and 0.91±0.30 ng/ml in AA, EA and non-athletes respectively, p = 0.09). Estradiol levels and the free androgen index (while lowest in AA) did not differ significantly across groups.

**Table 2:** Areal (DXA) and volumetric (HRpQCT) bone density measures, cortical microarchitectural parameters, and strength estimates (FEA) in amenorrheic athletes (AA), eumenorrheic athletes (EA) and non-athletes (NA).

	AA N = 38	EA N = 24	NA N = 23	ANOVA P-value	AA vs. EA P-value	AA vs. NA P-value	EA vs. NA P-value
**Spine aBMD (g/cm^2^)** †	0.91 (0.83–1.02)	0.99 (0.87–1.08)	0.96 (0.84–1.08)	**0.04** a	0.07^b^	0.12	1.00
**Spine aBMD Z-scores**	−0.75±1.25	0.03±0.98	−0.25±0.97	**0.02** a	**0.02** a	0.21	0.66
**Total hip aBMD (g/cm^2^)**	0.96±0.13	1.04±0.12	0.95±0.09	**0.01** a **^,^** c	**0.03** a **^,^** c	0.89	**0.02** a **^,^** c
**Total hip aBMD Z-scores**	0.05±1.10	0.81±0.90	−0.28±0.70	**0.0009** a **^,^** c	**0.01** a **^,^** c	0.40	**0.0009** a **^,^** c
**Femoral neck aBMD (g/cm^2^)** †	0.83 (0.76–0.93)	0.88 (0.82–0.98)	0.81 (0.74–0.89)	0.09^b^	0.16	0.91	0.12^b^ **^,^** d
**Femoral neck aBMD Z-scores**	−0.19±1.22	0.34±1.03	−0.59±0.80	**0.02** a **^,^** c	0.16	0.35	**0.01** a **^,^** c
**Whole body aBMD (g/cm^2^)** †	1.05 (0.99–1.12)	1.11 (1.04–1.18)	1.07 (0.99–1.13)	0.09^a^ **^,^** c	0.09^a^ **^,^** d	0.78	0.36^d^
**Whole body aBMD Z-scores**	−0.48±1.12	0.38±1.12	−0.39±0.99	**0.01** a **^,^** c	**0.01** a **^,^** c	0.95	**0.049** a **^,^** c
**Total vBMD (mgHA/cm^3^)**	294.0±62.2	305.8±51.4	332.3±72.9	0.08^b^	0.76	0.07^b^	0.33
**Trabecular vBMD (mgHA/cm^3^)** †	161.2 (134.5–177.5)	189.6 (155.3–203.4)	176.7 (149.2–203.4)	**0.048** b	0.095	0.12	0.93
**Cortical vBMD (mgHA/cm^3^)** †	923.7 (876.2–947.5)	934.1 (876.9–947.9)	950.8 (911.7–987.6)	**0.04** a **^,^** c	0.99	**0.046** a **^,^** c	0.13
**Total cross-sectional area (mm^2^)**	272.4±49.9	284.7±45.7	257.3±43.5	0.21	0.65	0.49	0.19
**% Cortical area**	19.6±5.0	18.8±3.8	22.9±4.5	**0.02** a **^,^** c	0.85	**0.04** a **^,^** d	**0.03** a **^,^** d
**Cortical thickness (mm)**	0.82±0.20	0.82±0.14	0.94±0.16	**0.04** b	0.99	**0.049** b **^,^** d	0.10
**Cortical porosity (%)**	1.10±0.78	0.70±0.44	0.66±0.40	**0.02** a **^,^** c	0.09^a^ **^,^** c	**0.04** a **^,^** c	0.98
**Stiffness (kN/m)** §	69.4 (57.2–88.2)	80.8 (73.5–86.5)	78.1 (68.4–92.3)	**0.03** a **^,^** d	0.11^b^ **^,^** d	**0.049** b	0.98
**Failure load (kN)** §	3.56 (2.97–4.52)	4.09 (3.73–4.40)	3.97 (3.53–4.61)	**0.03** a **^,^** d	0.10^b^ **^,^** d	**0.05** b	0.99

Mean ± SD or Median (Interquartile Range);

† Kruskal Wallis test;

§ p value reported for log converted data;

a p<0.05 after controlling for age,

b p<0.10 after controlling for age,

c p<0.05 after controlling for age and race (Black vs. non-Black),

d p<0.10 after controlling for age and race.

AA: amenorrheic athletes; EA: eumenorrheic athletes; NA: non-athletes; aBMD: area bone mineral density; vBMD: volumetric bone mineral density.

### Hormone Levels

**Figure 1:**
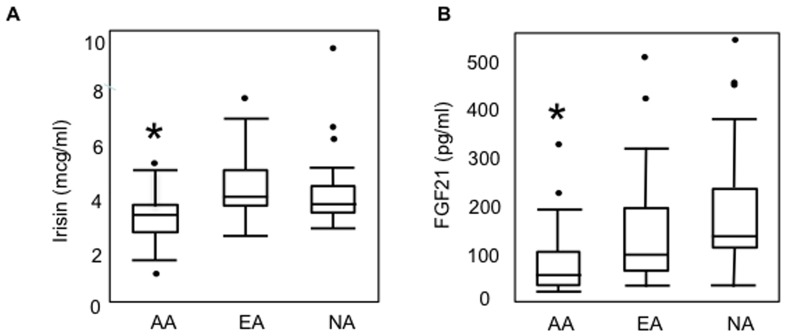
Irisin and FGF21 levels in athletes and non-athletes. (A) Irisin and (B) FGF21 levels were lower in amenorrheic athletes (AA) compared to eumenorrheic athletes (EA) and non-athletes (NA) (ANOVA for three-group comparison for log converted values, followed by the Tukey Kramer test to compare any two groups). *, p<0.05 vs. EA and NA.

Table 1 and Figure 1 demonstrate levels of irisin and FGF21 across the groups. Irisin and FGF21 levels were significantly lower in AA than EA and non-athletes, and these differences persisted after controlling for age (p = 0.0001 for both for the overall ANOVA), or for body fat and lean mass (p<0. 05), while there were no differences in hormone levels between EA and non-athletes. Log irisin and log FGF21 correlated positively with each other within athletes (r = 0.26, p = 0.04), but the association was inverse in non-athletes (r = −0.50, 0.01). Within individual athlete groups, associations were weaker, and mostly driven by AAs (AA: Spearman Rho = 0.28, p = 0.09; EA: Spearman Rho = 0.13, p = 0.54). Our results remained similar when we excluded the three athletes who had not attained menarche from the analysis.

### Relationship between Hormone Levels, Measures of Energy Expenditure and Measures of Body Composition

Associations of irisin with measures of energy expenditure and body composition are shown in Table 3. Irisin was positively associated with resting energy expenditure in the group as a whole, within athletes and also non-athletes. However, associations were not significant for AA and EA analyzed separately. We found no associations of irisin with hours/week of exercise or fat mass in any of the groups. Log irisin was positively associated with lean mass in all athletes and within AA, but not in EA, non-athletes or the group as a whole.

**Table 3:** Associations of irisin (log converted values) with areal (DXA) and volumetric (HRpQCT) bone density measures, cortical microarchitectural parameters, and strength estimates (FEA) in all subjects (All), amenorrheic athletes (AA), eumenorrheic athletes (EA), all athletes (ATH) and all non-athletes (NA).

	All		AA		EA		ATH		NA	
	r	p	r	p	r	p	r	p	r	p
**Energy Status and Body Composition**										
**Resting energy expenditure (calories)**	**0.39**	**<0.0001**	0.24	0.15	0.25	0.25	**0.46**	**0.0002**	0.49*	**0.02**
**Hours/week of exercise activity**	−0.10	0.34	0.20	0.23	0.27	0.22	0.18	0.16	0.07	0.74
**Lean mass (kg)**	−0.01	0.94	**0.34**	**0.04**	−0.09	0.67	0.26*	**0.04**	−0.07	0.76
**Fat mass (kg)**	0.03	0.76	0.11	0.51	−0.11	0.62	0.21	0.11	−0.37	0.08
**Bone Parameters**										
**Spine BMD Z-scores**	**0.21**	**0.03^a,b,c^**	0.03	0.84	0.28	0.19	**0.33**	**0.008^b,c^**	−0.20	0.35
**Total hip BMD Z-scores**	0.05	0.63	0.05	0.77	0.19	0.39	**0.31**	**0.02**	−0.11	0.6
**FN BMD Z-scores**	0.12	0.22	0.05	0.78	0.25	0.24	**0.30**	**0.02**	0.09	0.7
**Whole body BMD Z-scores**	**0.21**	**0.04^a,b,c^**	0.18	0.30	0.11	0.63	**0.36**	**0.005^a,b,c^**	0.24	0.27
**Total vBMD (mgHA/cm^3^)**	**0.30**	**0.007^a^**	0.25	0.15	0.43*	**0.04^a,b^**	**0.28**	**0.03**	0.14	0.53
**Trabecular vBMD (mgHA/cm^3^)**	**0.31**	**0.005^a^**	0.10	0.54	0.38	0.08	**0.30**	**0.02**	0.23	0.30
**Cortical vBMD (mgHA/cm^3^)**	0.16	0.19	0.24	0.18	0.04	0.87	0.18	0.2	−0.18	0.44
**Total cross-sectional area (mm^2^)**	0.07	0.58	0.07	0.69	0.17	0.51	0.15	0.29	0.03	0.90
**Cortical area/total area**	0.15	0.21	0.08	0.66	0.27	0.30	0.06	0.55	0.02	0.90
**Cortical thickness (mm)**	0.18	0.13	0.09	0.60	0.51*	**0.04^a^**	0.14	0.33	0.08	0.70
**Cortical porosity (%)**	−0.16	0.18	−0.23	0.18	−0.04	0.87	−0.23	0.10	0.18	0.44
**Stiffness (kN/m)**	**0.34**	**0.004^a,b^**	0.25	0.15	0.32	0.22	**0.37**	**0.007**	0.12	0.63
**Failure load (kN)**	**0.34**	**0.003^a,b,c^**	0.23	0.18	0.26	0.32	**0.38**	**0.006**	0.13	0.59
**P1NP (ng/ml)**	0.05	0.71	−0.16	0.53	0.02	0.95	−0.12	0.47	0.60*	**0.008^a,b,c^**
**CTX (ng/ml)**	−0.03	0.84	0.06	0.82	−0.31	0.20	−0.13	0.46	0.16*	0.52

Spearman correlations; *Pearson correlations;

For bone parameters: ^a^p<0.05 after controlling for lean mass; ^b^p<0.05 after controlling for lean mass and activity; ^c^p<0.05 after controlling for lean mass, activity and fat mass; BMD: bone mineral density; FN: femoral neck; vBMD: volumetric bone mineral density.

All: all subjects; AA: amenorrheic athletes; EA: eumenorrheic athletes; ATH: all athletes; NA: non-athletes.

P values ≤0.05 are bolded.

Associations of FGF21 with measures of energy expenditure, body composition are shown in Table 4. Log FGF21 was associated inversely with resting energy expenditure in EA and non-athletes, but not in the other groups. Across all groups, there were negative associations between log FGF21 and with hours/week of exercise, and positive associations with body fat content. Within individual groups, we found no associations of log FGF21 with hours/week of exercise, or percent body fat mass. There were no associations of log FGF21 with lean mass. Irisin and FGF21 levels were overall not associated with age (p = 0.52 and 0.85 respectively), or bone age (p = 0.89 and 0.61).

**Table 4:** Associations of FGF-21 (log converted values) with areal (DXA) and volumetric (HRpQCT) bone density measures, cortical microarchitectural parameters, and strength estimates (FEA) in all subjects (All), amenorrheic athletes (AA), eumenorrheic athletes (EA), all athletes (ATH) and all non-athletes (NA).

	All		AA		EA		ATH		NA	
	r	p	r	p	r	p	r	p	r	p
**Energy Status and Body Composition**										
**Resting energy expenditure (calories)**	−0.04	0.72	0.16	0.35	−**0.40**	**0.05**	0.19	0.14	−**0.47**	**0.02**
**Hours/week of exercise activity**	0.04	0.70	−0.09	0.58	−0.02	0.94	−0.05	0.71	−0.05	0.83
**Lean mass (kg)**	0.02	0.86	−0.01	0.96	−0.34	0.10	−0.05	0.72	−0.20	0.36
**Fat mass (kg)**	**0.34**	**0.0004**	0.16	0.34	0.01	0.95	0.22	0.09	0.32	0.14
**Bone Parameters**										
**Spine BMD Z-scores**	0.07	0.48	−0.01	0.94	0.02	0.94	0.15	0.25	−0.06	0.80
**Total hip BMD Z-scores**	0.03	0.76	−0.04	0.80	0.04	0.85	0.14	0.28	0.04	0.85
**FN BMD Z-scores**	0.04	0.72	0.03	0.87	0.003	0.99	0.15	0.23	−0.12	0.58
**Total vBMD (mgHA/cm^3^)**	0.04	0.73	−0.01	0.94	0.14	0.51	0.09	0.52	−**0.45**	**0.03** a **^,^** b **^,^** c
**Trabecular vBMD (mgHA/cm^3^)**	0.006	0.96	−0.05	0.77	0.09	0.69	0.11	0.40	−**0.52**	**0.01** a **^,^** b **^,^** c
**Cortical vBMD (mgHA/cm^3^)**	**0.25**	**0.04**	0.20	0.26	0.12	0.64	0.17	0.23	0.04	0.87
**Total cross-sectional area (mm^2^)**	−0.06	0.64	−0.03	0.86	−0.07	0.79	0.03	0.86	0.08	0.73
**Cortical area/total area**	0.04	0.71	−0.11	0.54	0.20	0.43	−0.03	0.84	−0.24	0.31
**Cortical thickness (mm)**	0.08	0.49	−0.11	0.53	0.27	0.29	0.01	0.94	−0.27	0.25
**Cortical porosity (%)**	−0.24*	**0.04^a^**	−0.03	0.84	−0.36	0.16	−0.16	0.25	−0.11	0.63
**Stiffness (kN/m)**	0.099	0.41	0.005	0.98	0.03	0.90	0.15	0.28	−0.30	0.19
**Failure load (kN)**	0.09	0.43	−0.01	0.95	0.06	0.82	0.15	0.29	−0.27	0.26
**P1NP (ng/ml)**	−0.07	0.62	0.11	0.67	−0.21	0.39	−0.05	0.77	−0.06	0.80
**CTX (ng/ml)**	0.05	0.71	−0.04	0.86	0.14	0.58	0.11	0.51	0.08	0.75

Spearman correlations;

a p<0.05 after controlling for lean mass;

b p<0.05 after controlling for lean mass and activity;

c p<0.05 after controlling for lean mass, activity and fat mass; BMD: bone mineral density; FN: femoral neck; vBMD: volumetric bone mineral density.

All: all subjects; AA: amenorrheic athletes; EA: eumenorrheic athletes; ATH: all athletes; NA: non-athletes.

P values ≤0.05 are bolded.

### Relationship between Hormone Levels and Sex Steroids

Log irisin levels were associated positively with the free androgen index in AA (r = 0.63, p = 0.002), and with estradiol in non-athletes (rho = 0.46, p = 0.05). Log FGF21 was associated positively with the free androgen index in AA (r = 0.55, p = 0.009) and inversely with estradiol levels in non-athletes (r = −0.63, p = 0.005). Associations of irisin and FGF21 with the free androgen index (but not estradiol) remained significant after controlling for fat mass or lean mass.

### Relationship between Hormone Levels and Bone Parameters

Across all groups, irisin levels were positively associated with spine, femoral neck and whole body BMD Z scores (Table 3). Irisin levels were also associated with total and trabecular vBMD, and with bone strength estimates, including stiffness and failure load. All associations appeared to be driven mostly by the athletes taken together (AA and EA). Associations within AA and EA groups were weaker. Table 3 also shows associations that remained significant after controlling for (i) lean mass, (ii) lean mass and activity levels, and (iii) lean mass, fat mass and activity levels. In contrast, the only associations observed between FGF21 and bone parameters were a positive association with cortical volumetric BMD, and an inverse association with cortical porosity for all groups taken together, and inverse associations with total and trabecular vBMD in non-athletes.

We tested for interactions between subject groups and irisin and FGF21 in determining associations of irisin with bone parameters across groups, and our analysis did not show strong evidence that the patterns of irisin effects on bone outcomes were different across subgroups.

Across all groups, the association of irisin with whole body BMD Z-scores, total and trabecular vBMD, percent cortical area, cortical thickness, stiffness and failure load were significant after controlling for FGF21 and lean mass (p = 0.01, 0.04, 0.02, 0.05, 0.04, 0.02, 0.02 and 0.04 respectively). In addition, spine and whole body BMD Z-scores, stiffness and failure load remained significantly associated with irisin after controlling for FGF21, lean mass and hours/week of exercise (p = 0.03, 0.01, 0.05 and 0.04 respectively), and for FGF21, lean and fat mass and hours/week of exercise (p = 0.04, 0.01, 0.06 and 0.04 respectively). Within athletes, the association between irisin and spine and whole body BMD Z-scores remained significant after controlling for FGF21, lean and fat mass, and hours/week of exercise. A trend persisted for the negative association between FGF21 and cortical porosity after controlling for irisin and lean mass (p = 0.08), but not after controlling for hours/week of activity and/or fat mass. Our results were similar when we excluded the three premenarchal athletes from our analysis.

## Discussion

This is the first study to demonstrate low levels of irisin and FGF-21 in adolescent amenorrheic athletes, which we speculate represents an adaptive response to conserve energy in these young women. Irisin levels were associated with resting energy expenditure and FGF-21 with body fat content. Importantly, irisin levels were associated with measures of areal and volumetric bone density and strength estimates in athletes, and associations with bone density and strength measures persisted after controlling for potential confounders. In contrast, FGF21 levels were associated with only a few bone parameters.

Irisin and FGF-21 promote the “browning” of white adipose tissue, resulting in “beige fat”, a metabolically favorable fat capable of burning energy through thermogenesis. This specialized tissue has features of brown fat, including multilocular fat cells, an abundance of mitochondria, and UCP-1, an enzyme that uncouples oxidative phosphorylation from ATP production, leading to energy release as heat [2], [4].

Irisin levels have not been previously reported in adolescent female athletes. One study suggested that in moderately trained young healthy male athletes, plasma irisin levels are increased 30 minutes after sprinting, but this effect is not seen after eight weeks of training [3]. In another study, initiation of an endurance exercise routine in sedentary men led to a two-fold rise in plasma irisin levels at ten weeks [2], while a third study in adults reported an increase in irisin following acute exercise, but a decrease in irisin after a 12-week period of endurance and strength training [26]. We found that irisin levels were lower in AA compared to EA and non- athletes, and these differences persisted after controlling for fat and lean mass. Our data likely reflect the effects of chronic exercise on irisin levels, in contrast to some previous studies that examined effects of short-durations of exercise on irisin secretion. Our data are consistent with a study of adult men that reported inverse associations of irisin with regular exercise related physical activities [27]. Furthermore, irisin levels were positively associated with resting energy expenditure. Irisin levels did not differ between EA and non-athletes in our study, suggesting that chronic exercise does not affect irisin levels in the setting of eumenorrhea and preserved energy stores. Decreased irisin levels in AA likely represent an adaptive response in chronic exercisers to reduce REE and conserve energy by reducing brown adipogenesis. This is corroborated by reports of lower irisin levels [25] and lower occurrence of brown fat in conditions of extreme malnutrition, such as anorexia nervosa [28], reductions in circulating irisin following marked weight loss in patients after bariatric surgery [22], and increased levels in obesity [25].

We did not assess brown fat in our subjects, and this is an area of future study. Of note, although our AA subjects had lower BMI than EA, their BMI was not as low as in girls with anorexia nervosa. Thus, these AA subjects are in a state of subtle energy deficit as indicated by their reduced fat mass and lower resting energy expenditure after correcting for lean mass, but not in the state of extreme energy deficit observed in anorexia nervosa. Another small study in patients with anorexia nervosa assessed irisin levels in those reporting moderate vs. high levels of physical activity, and found no differences in BMI, REE or irisin levels across groups, although the higher activity group had higher total energy expenditure [29]. These data suggest that irisin drives changes in REE (or vice versa), but not in total energy expenditure, consistent with irisin being an inducer of brown adipogenesis (and driver of REE), and the positive associations of irisin with REE in our subjects.

In addition, consistent with irisin being a myokine and with other studies that have reported positive associations of irisin with muscle mass [22], [25], [30], we observed positive associations of irisin with lean mass in athletes. Other studies have reported positive associations of irisin with nutritional markers such as BMI, fat mass, IGF-1 and insulin, and with markers of the metabolic syndrome such as higher blood pressure, insulin resistance and lipid levels, [22], [25], [27]. Higher irisin levels in obesity, and in patients with the metabolic syndrome may represent a physiological response to improve glucose tolerance and lipid parameters in these individuals [25], [26], [27]. One study reported lower irisin levels in patients with type 2 diabetes than controls of similar BMI, which may represent a failure of this physiological response leading to diabetes [31]. However, a study in obese children reported an increase (rather than a decrease in irisin levels after a year of exercise and lifestyle intervention [32]. These seemingly conflicting data speak to the complexity of irisin interactions with metabolic endpoints, and the need for further studies to clarify the role of irisin as a regulator of metabolism. We found no associations of irisin with fat mass in our study, maybe because we assessed only lean individuals in our cohort.

Of interest, irisin levels were higher in our study than reported in many previous studies, likely consequent to differences in the specific assay used for analysis [20] and the younger age of our subjects. Some studies have reported inverse associations of irisin with age [22], [33], and several fold higher irisin levels in young adult men compared with middle aged women [22].

Moreover, irisin has a potential role in modulating bone metabolism through direct [10], [34] and indirect (brown fat mediated) effects. A recent study demonstrates that irisin may affect osteoblastogenesis via the Wnt/beta-catenin pathway downstream of the BMP receptor signal, and also inhibits osteoclast differentiation [10]. Furthermore, *in vivo* studies indicate that irisin increases bone trabecular volume and cortical thickness in mice [10]. Our data are consistent in that irisin levels predicted trabecular volumetric BMD measures in addition to other measures of bone density and strength estimates in athletes. Effects of irisin on bone may also be mediated via induction of brown adipogenesis given reports of associations of brown fat with bone strength estimates in women [8], and with cortical thickness in children and adolescents [9]. In adult women, lower irisin levels have been associated with a higher risk of osteoporotic fractures [33].

Similar to irisin, FGF21 levels increase with acute or short durations of exercise [35], [36], decrease with regular exercise and caloric restriction in animals and humans [37], [38], decrease in extreme states of malnutrition (as in anorexia nervosa) related to BMI [24], and increase in obesity and the metabolic syndrome related to fat mass [39], [40], [41]. Given known effects of FGF21 in inducing brown adipogenesis [4], [5] and increasing REE, these changes likely represent an adaptive response to decrease brown adipogenesis and conserve energy in anorexia nervosa, and to increase brown adipogenesis and expend energy in obesity. Conversely, these changes may also signal an FGF21 resistant state. Consistent with the study in women with anorexia nervosa [24], we found that FGF21 levels were low in AA compared to EA and non athletes and inversely associated with hours per week of exercise, likely again an adaptive response to conserve energy. In contrast, a study of sedentary healthy young women reported an increase in FGF-21 levels after two-weeks of exercise [24], suggesting that short-term energy expenditure in sedentary women may result in increases in FGF-21 whereas longer-term chronic energy expenditure leads to decreased FGF-21 to preserve energy. FGF21 also impacts bone metabolism, although available reports are contradictory with some studies indicating beneficial [12], [13], and others indicating deleterious [11] effects of FGF21 on bone. In our study, FGF21 was associated positively with cortical volumetric BMD and inversely with cortical porosity for the group taken together, consistent with a beneficial effect of FGF21 on bone. However, associations were lost after controlling for irisin, body composition and exercise activity. Further studies are needed to clarify effects of FGF-21 on bone.

Few studies have examined associations of irisin and FGF21 with the sex steroids. We observed positive associations of both hormones with the free androgen index in AA. However, associations of these hormones with estradiol were conflicting, with positive associations noted with irisin and inverse associations with FGF21 in non-athletes. Of interest, positive associations of FGF21 with testosterone levels have been reported in women with polycystic ovarian syndrome (PCOS) [42], and positive associations of irisin and FGF21 with testosterone levels may reflect associations of these hormones with components of the metabolic syndrome. Although one study reported higher FGF21 levels in women with PCOS than in those without this condition, another study found no differences in FGF21 across groups [43].

## Conclusions

We report low levels of irisin and FGF-21 in young amenorrheic athletes compared with eumenorrheic athletes and non-athletes, even after taking into account fat and lean mass. In addition, we demonstrate that irisin levels are a determinant of measures of areal and volumetric bone mineral density and bone strength estimates in athletes, but not non-athletes. While this is the first study to report levels of irisin and FGF21 in adolescent female athletes and non-athletes, it is a cross-sectional study and therefore causality cannot be inferred from our data. Further research regarding the role of these hormones in regulating bone metabolism will be important.
